# Genomic Characteristics of Elite Maize Inbred Line 18-599 and Its Transcriptional Response to Drought and Low-Temperature Stresses

**DOI:** 10.3390/plants11233242

**Published:** 2022-11-25

**Authors:** Yang Cao, Jingtao Qu, Haoqiang Yu, Qingqing Yang, Wanchen Li, Fengling Fu

**Affiliations:** 1 Maize Research Institute, Sichuan Agricultural University, Chengdu 611130, China; 2 CIMMYT-China Specialty Maize Research Center, Crop Breeding and Cultivation Research Institute, Shanghai Academy of Agricultural Sciences, Shanghai 201403, China

**Keywords:** drought, genomics, low temperature, maize, transcriptomics

## Abstract

Elite inbred line 18-599 was developed via triple test cross from introduced hybrid P78599 and used as parents of dozens of maize hybrids adapting to the diverse ecological conditions of the maize ecological region in Southwest China. In this study, its genomic DNA was resequenced and aligned with the B73 genome sequence to identify single nucleotide polymorphism (SNP), and insertion (In) and deletion (Del) loci. These loci were aligned with those between B73 and 1020 inbred lines in the HapMap database to identify specific variation loci of 18-599. The results showed that there were 930,439 specific SNPs and 358,750 InDels between 18-599 and the 1020 lines. In total, 21,961 of them showed significant impacts on the functions of 12,297 genes, such as frameshift, change of splicing site, stop gain, change of start site, and stop loss. Phylogenetic analysis showed that 18-599 was closely related to inbred lines ZEAxujRAUDIAAPE and 2005-4, but far from some inbred lines directly isolated from P78599. This result indicated that 18-599 not only pyramided the elite genes of P78599, but also acquired genetic divergence during the repetitive backcrosses of triple test cross to confer its elite agronomic characteristics. Subsequently, the RNA of 18-599 was sequenced. The aligned 9713 and 37,528 of the 165,098 unigenes were screened and aligned with annotated transcripts of the B73 genome differentially expressed under drought and low-temperature stress, respectively, and their functions were involved in the responses to these stresses. The quantitative PCR results of fourteen random genes verified the RNA sequencing results. These findings suggest that the transcriptional responses of many resistance-related genes were an important mechanism for 18-599 to adapt to diverse ecological conditions.

## 1. Introduction

The conventional method of maize breeding is to isolate homozygous plants from germplasm resources and develop inbred lines with elite agronomic characteristics, wide adaptability, good quality, and high reproductive capacity. These inbred lines are evaluated for their combining ability by test cross and used as parents of hybrids. After evaluation for their high yield, adaptability, and quality by multiyear and multilocation experiments, superior hybrids are approved for commercial dissemination [1,2,3,4]. Therefore, the germplasm resources present the most fundamental source of elite agronomic characteristics of these superior hybrids.

Maize is originally an allogamous plant. After long-term domestication and selection under natural and cultivation conditions, abundant genetic divergences have accumulated, and a variety of germplasm resources with different characteristics have been inherited. However, the breeding and dissemination of hybrids between inbred lines have failed to protect germplasm resources while doubling maize yield. As a result, the germplasm resources available for further improvement of maize are becoming increasingly scarce. Most of the parents of the current commercial hybrids are “second cycle lines” isolated from the parents of the disseminated hybrids of the last cycle. Because of their close genetic relationships, not only has further improvement become increasingly difficult, large-scale production reduction caused by sudden diseases and insect pests is also becoming a potential threat [5,6,7,8,9,10].

In order to overcome these challenges brought by scarce germplasm resources, Chinese breeders and researchers have been diligently investigating methods for germplasm enhancement since the 1980s. Commercial hybrids, germplasm populations, and inbred lines were introduced from the United States and some other countries in temperate, tropical and subtropical zones. After domestication and improvement, numerous inbred lines have been isolated from them or their crosses with domestic germplasm and used as parents of many commercial hybrids, which have been disseminated extensively [9,11,12,13,14]. From the introduced hybrid P78599, a series of elite inbred lines, such as Qi319, CAU178, P138, Duohuang 29, Dan 598, Dan 599, Shen 137, Shen 135, R150, Yu 87-1, Cheng 18, Shan 89-1, Han 21, and 78599, have been isolated and used as parents of many commercial hybrids, such as Nongda 108, Ludan 981, Nongda 3138, Yuyu 22, Danyu 23, Danyu 24, Danyu 26, and Shanzi 1, which have provided great benefits for maize production in China [12,15]. On the other hand, most of these inbred lines also exhibit certain disadvantages, such as tall plant, high ear position, developed tassel, long growth period, and photoperiod sensitivity, due to the tropical germplasm of P78599 [16].

In view of the diverse ecological conditions of ecological maize regions in Southwest China, such as seasonal drought, conspicuous three-dimensional and rainy climate, serious diseases, and extensive cultivation, inbred line 18-599 was developed using the triple test cross method from P78599, and evaluated under diverse ecological conditions [17]. Owing to its high combining ability, high reproduction capacity, strong resistance, extensive adaptability, and elite agronomic characteristics, it has been used as the parents of dozens of commercial hybrids, such as Chuandan 13, Dong 315, and Zunyu 8, which have been approved by the national government and disseminated over more than ten-million hectares in this region. Inbred line 18-599 was awarded the secondary prize of technological invention by the Chinese central government [18,19].

In the present study, the genomic DNA of 18-599 was resequenced, and aligned with B73 genome sequence to identify single nucleotide polymorphism (SNP), insertion (In) and deletion (Del) loci. Subsequently, these loci were aligned with those between B73 and 1020 inbred lines in the HapMap database v3.21, to identify specific variation loci of 18-599 and their functional annotations. Based on their identical variation loci, a phylogenetic tree was conducted among 18-599 and the 1020 lines to analyze their genetic relationships. The differential expression of 18-599 seedlings under drought and low-temperature stress was detected by RNA sequencing (RNA-Seq) and verified by real-time quantitative PCR (RT-qPCR) to illuminate the molecular mechanism of 18-599 regarding its strong resistance and extensive adaptability to diverse ecological conditions.

## 2. Results

### 2.1. Genomic Variation of 18-599

After quality evaluation and base calling, the raw files of resequencing were converted into FASTQ format of 407,720,096 raw reads (61.16 Gb). The resequencing coverage was estimated as 29×. After removing 3,685,080 (0.9%) adaptor contaminations, 1,653,706 (0.41%) low quality, and 51,164 (0.01%) unknown bases n > 1%, a total of 402,330,146 clean reads (60.35 GB) were obtained, accounting for 98.68% of the raw reads.

**Figure 1:**
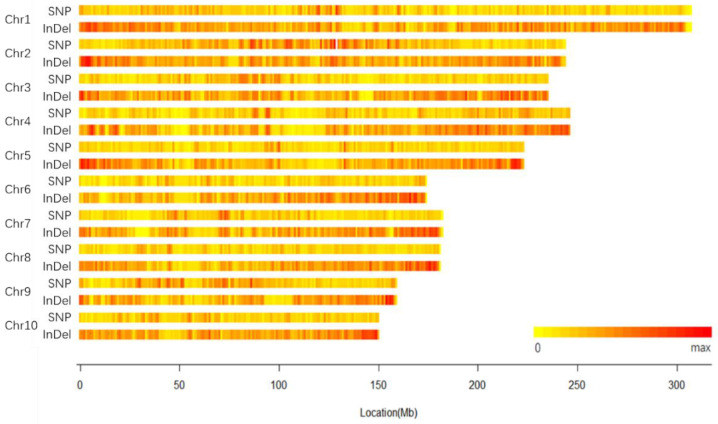
Distribution heatmap of SNPs and InDels between 18-599 and reference genome.

Of the 402,330,146 clean reads, 359,628,515 were mapped to the maize reference genome B73 (v4), accounting for 89.39%. After removing the duplicates of PCR amplification and varication calling, 4,713,972 variation loci [4,125,020 SNPs and 588,952 InDel (285,503 Insertion and 303,449 Deleltion)] were called by Genome Analysis Toolkit software (GATK). The length of the InDels varied from 1 to 50 bp. The distribution of these SNPs and InDels on the ten maize chromosomes is shown in Figure 1. Their average distribution was 1933 SNPs/Mb and 276 InDels/Mb, respectively.

### 2.2. Specific Variation Loci of 18-599

Of the 4,125,020 SNPs and 588,952 InDels called by the alignment between genomic sequences of inbred lines 18-599 and B73, 3,213,863 SNPs and 230,202 InDels were matched with the allelic variation loci aligned between B73 and the 1020 inbred lines in the HapMap database (v3.21). The other 911,157 SNPs and 358,750 InDels were identified as non-allelic specific loci of 18-599. Of the matched SNPs and InDels, the genotypes of 19,282 loci were found to be different from those aligned between B73 and the 1020 inbred lines and identified as specific genotypes of 18-599. Of the total 930,439 specific SNPs and 358,750 specific InDels between 18-599 and the 1020 lines, 21,961 loci showed significant impacts on the functions of 12,297 genes, such as frameshifts, change of splicing sites, stop gains, change of start sites, and stop losses (Table 1).

### 2.3. Identical Variation Loci and Phylogenetic Tree

**Figure 2:**
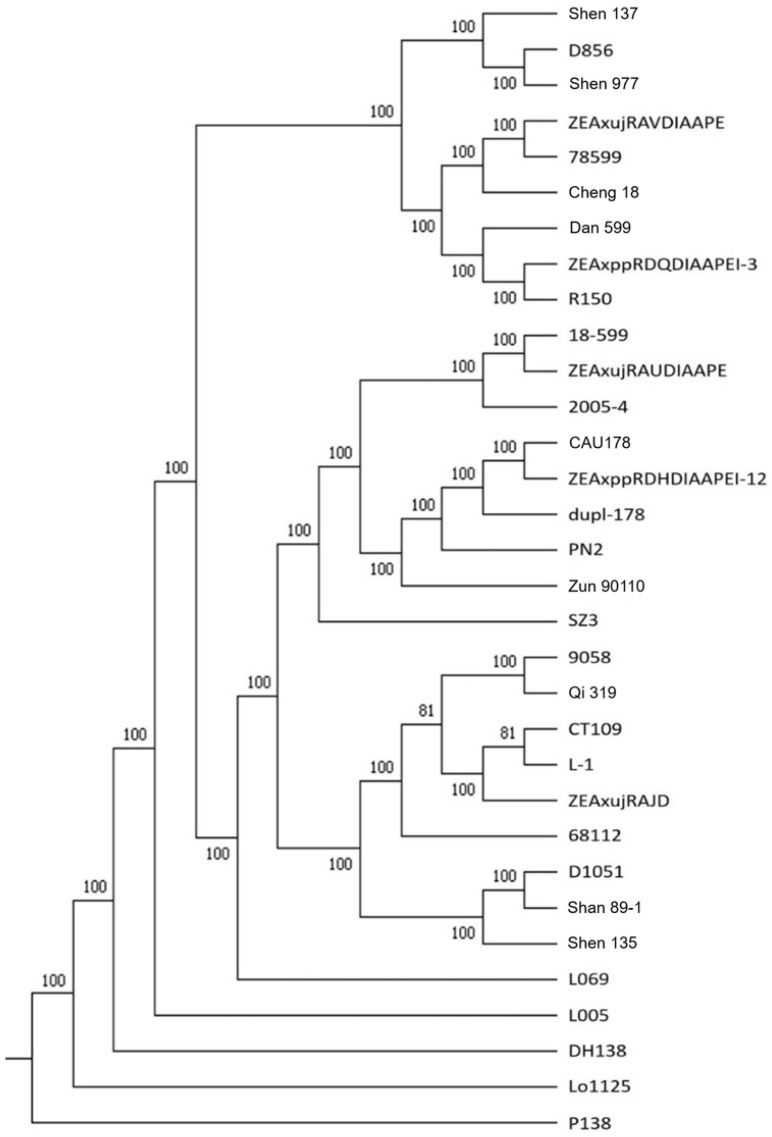
Phylogenetic tree among 18-599 and 31 inbred lines.

The alignment showed that 18-599 shared 30–40% identical variation loci with 743 of the 1020 inbred lines, and more than 50% identical variation loci with 31 of them (Table 2). After removing the deletion and heterozygous genotypes, these 31 inbred lines were used to construct a phylogenetic tree with 18-599 (Figure 2). The result showed that 18-599 was closely related to inbred lines ZEAxujRAUDIAAPE and 2005-4, and CAU 178 isolated from hybrid P78599, as well as 78,599 itself, but situated far from inbred lines Cheng 18, Dan 599, R150, and other inbred lines isolated from hybrids P78599 and P78641.

### 2.4. Transcriptional Response to Drought and Low-Temperature Stress

From the reversely transcribed cDNA samples, 489,185,064, 63,318,174, and 58,323,172 raw reads were obtained from the drought-treated samples, 69,540,300, 76,434,556, and 56,314,974 raw reads from the low-temperature treated samples, and 58,054,200, 62,959,680 and 64,099,664 raw reads from the blank control samples, respectively. After removing those with adaptor contamination, low-quality and unknown base >1%, 67,129,352, 65,382,452 and 54,511,976, 56,384,548, 61,726,740 and 65,417,140, and 48,021,670, 61,820,870 and 56,798,602 clean reads were retained, respectively, accounting for more than 96% of the raw reads. About 70% of the clean reads were mapped to the B73 genomic sequence (Table S1). From the unmapped, a total of 165,098 unigenes with a total length of 74,602,848 bp and a contig N50 of 496 bp were assembled by TRINITY.

By using Stringtie and DESeq v2. 2, 12,914 differentially expressed genes (DEGs) were identified in response to drought stress (Table S2). Among them, the expressions of 6630 genes were up-regulated with an average multiple (log_2_) of 2.33 times. The up-regulated multiple (log_2_) of gene *Zm00001d015053* was as high as 24.35 times. The expressions of 6374 genes were down-regulated with an average multiple (log_2_) of 2.44 times. The down-regulated multiple (log_2_) of gene *TRINITY_ DN38188_ c10_ g1* was as low as 21.18 times. A total of 46,698 differentially expressed unigenes were identified in response to low-temperature stress (Table S3). Among them, the expressions of 21,532 genes were up-regulated, with an average multiple (log_2_) of 2.48 times. The upregulated multiple (log_2_) of gene *TRINITY_DN75068_c0_g1* was as high as 22.61 times. The expressions of 25,252 genes were down-regulated, with an average multiple (log_2_) of 2.87 times. The down-regulated multiple (log_2_) of gene *Zm00001d038996* was as low as 143.89 times.

### 2.5. RT-qPCR Verification

**Figure 3:**
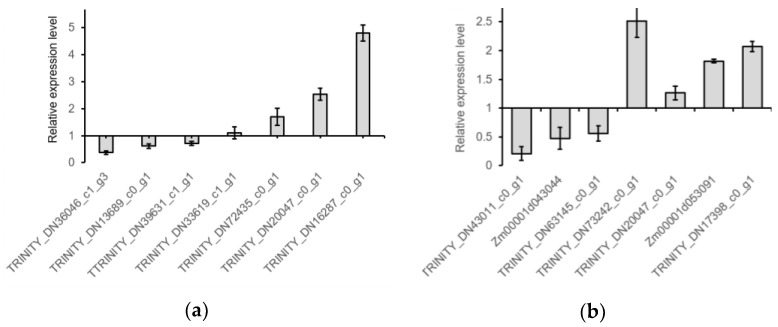
The relative expression levels of fourteen genes in response to drought and low-temperature stress. (**a**) Seven genes in response to drought stress; (**b**) Seven genes in response to low-temperature stress.

The RT-qPCR results showed that the relative expression levels of genes *TRINITY_DN36046_c1_g3*, *TRINITY_DN13689_c0_g1* and *TTRINITY_DN39631_c1_g1* were significantly down-regulated, and those of genes *TRINITY_DN33619_c1_g1*, *TRINITY_DN72435_c0_g1*, *TRINITY_DN20047_c0_g1* and *TRINITY_DN16287_c0_g1* were significantly up-regulated in response to drought stress (Figure 3a). In response to low-temperature stress, the relative expression levels of genes *TRINITY_DN43011_c0_g1*, *Zm00001d043044*, and *TRINITY_DN63145_c0_g1* were significantly down-regulated, and those of genes *TRINITY_DN73242_c0_g1*, *TRINITY_DN20047_c0_g1*, *Zm00001d053091*, and *TRINITY_DN17398_c0_g1* were significantly up-regulated (Figure 3b). These results verify the above transcriptomics analysis results.

### 2.6. Functional Annotation of Differentailly Expressed Genes

**Figure 4:**
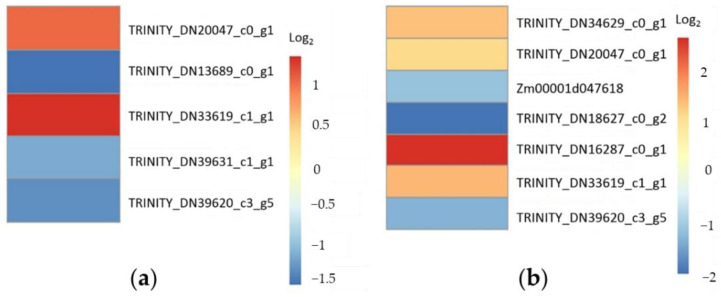
Differential expression heatmap of twelve genes in response to drought stress. (**a**) Log_2_ value heatmap of five differentially expressed genes in response to water deprivation (GO: 0009414); (**b**) Log_2_ value heatmap of seven differentially expressed genes in response to osmotic (GO: 0006970) and salt (GO: 0009651) stresses.

**Figure 5:**
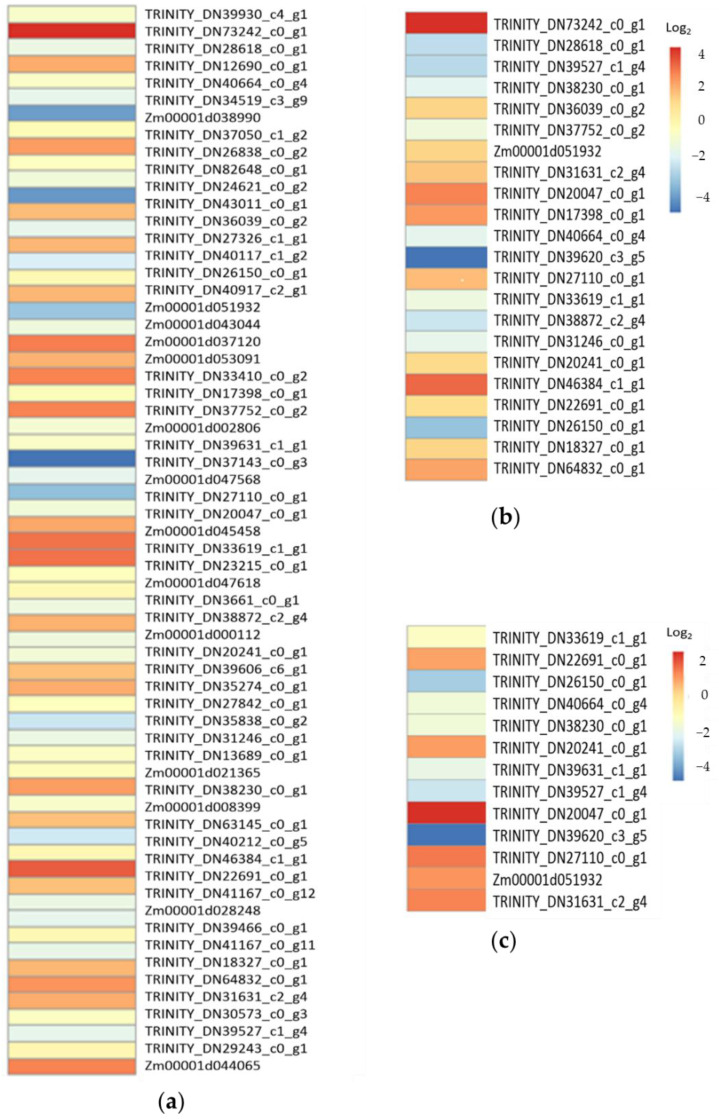
Differential expression heatmap of 102 genes in response to low-temperature stress. (**a**) Log_2_ value heatmap of 66 differentially expressed genes in response to abiotic stimuli (GO: 0009628); (**b**) Log_2_ value heatmap of 23 differentially expressed genes in response to temperature stimulus (GO: 0009266); (**c**) Log_2_ value heatmap of 13 differentially expressed genes in response to cold stress (GO: 0009409).

Under drought stress, 855 of the 12,914 differentially expressed genes were annotated in the CDS of B73 genomic sequence, Non-Redundant Protein Sequence (NR) Database and its annotation, as well as Gene Ontology (GO) annotation (Table S2). They were involved in biological processes (475), molecular functions (214), and cell components (95). Among them, genes *TRINITY_DN20047_c0_g1* and *TRINITY_DN33619_c1_g1* upregulated 2.17 and 2.67 times, and genes *TRINITY_DN13689_c0_g1*, *TRINITY_DN39631_c1_g1*, and *TRINITY_DN39620_c3_g5* downregulated 2.93, 2.27, and 2.54 times, respectively, were classified as responsive to water deprivation (GO: 0009414) (Figure 4a). Genes *TRINITY_DN20047_c0_g1*, *TRINITY_DN34629_c0_g1*, *TRINITY_DN33619_c1_g1*, and *TRINITY_DN16287_c0_g1* upregulated 2.17, 2.56, 2.67, and 6.13 times, and genes *TRINITY_DN18627_c0_g2*, *TRINITY_DN39620_c3_g5*, and *Zm00001d047618* downregulated 4.10, 2.54, and 2.27 times, respectively, were classified as responsive to osmotic stress (GO: 0006970) and salt stress (GO: 0009651) (Figure 4b). Under low-temperature stress, 1394 of the 37,528 differentially expressed genes were annotated (Table S3) and involved in biological processes (845), molecular functions (368), and cell components (181). Among them, 65 were classified as responsive to abiotic stimuli (GO: 0009628) (Figure 5a), 22 as responsive to temperature stimulus (GO: 0009266) (Figure 5b), and 13 as responsive to cold stress (GO: 0009409) (Figure 5c).

## 3. Discussion

The resequencing and genomics analysis showed that elite inbred line 18-599 had 21,961 specific variation loci in 12,297 genes while aligned against the 1020 inbred lines in the HapMap database (Table 1). The phylogenetic analysis showed that 18-599 was closely related to ZEAxujRAUDIAAPE and 2005-4, as well as CAU 178 isolated from hybrid P78599, but situated far from 78599, Cheng18, Dan 599, R150, and other inbred lines isolated from hybrids P78599 and 78641 (Table 2 and Figure 2). This result indicated that 18-599 was isolated from the breeding population constructed with hybrids P78599 and P78641 by the triple test cross method and evaluated under diverse ecological conditions [17], which not only inherited some elite germplasm of P78599 and P78641 but also accumulated abundant genetic variation with 78599, Cheng 18, Dan 599, R150 and some other lines isolated from the same hybrids by conventional methods, involving many genes responding to drought and low-temperature stress (Figure 3 and Figure 4). The elite genotypes of these genes were pyramided by the repetitive test crosses and backcrosses of the triple test cross and conferred high combining ability, high reproduction capacity, strong resistance, extensive adaptability, and elite agronomic characteristics of 18-599 [18,19].

RNA-Seq and transcriptomics analysis showed that the expressions of 9713 genes were responsive to drought stress. Of these, 855 were annotated and involved in 475 biological processes (475), molecular functions (214), and cell components (95). The functional classification of five was responsive to water deprivation, and seven was responsive to osmotic and salt stresses (Figure 4). Among them, the expression of genes *Zm00001d015053* and *TRINITY_DN38188_c10_g1* were upregulated 24.35 and downregulated 21.18 times, respectively. They encode terpene synthase 2 and a transmembrane cationic amino acid transporter protein, respectively. The relative expression levels of seven randomly sampled genes detected by RT-qPCR verified the results of RNA-Seq and transcriptomics analysis (Figure 3a). The three down-regulated genes *TRINITY_DN36046_c1_g1*, *TRINITY_DN13689_c0_g1*, and *TRINITY_DN39631_c1_g1* encode S1 RNA binding domain, a sterol desaturase, and a magnesium-dependent ATP hydrolase, respectively. The four up-regulated genes *TRINITY_DN33619_c1_g1*, *TRINITY_DN72435_c0_g1*, *TRINITY_DN20047_c0_g1*, and *TRINITY_DN16287_c0_g1* encode a lipopolysaccharide kinase, a heat shock factor, an annexin, and a BetVI protein, respectively. The orthologs or paralogs of these nine genes were well documented for their response to drought, salt, and diverse abiotic stresses [20,21,22,23,24,25,26,27,28,29,30,31,32,33,34,35,36].

The expressions of 37,528 genes were responsive to low-temperature stress. Of these, 1394 were annotated and involved in biological processes (845), molecular functions (368), and cell components (181). The function classification of 65 was responsive to abiotic stimulus, 22 responsive to temperature stimulus, and 13 responsive to cold stress (Figure 5). Among them, the expression of gene *TRINITY_DN75068_c0_g1* was upregulated 22.61 times. Its function has not been annotated. The expression of gene *Zm00001d038996* was downregulated 143.89 times. It encodes glycosyltransferase 1. The relative expression levels of seven randomly sampled genes detected by RT-qPCR verified the results of RNA-Seq and transcriptomics analysis (Figure 3b). The three down-regulated genes *TRINITY_DN43011_c0_g1*, *Zm00001d043044*, and *TRINITY_DN63145_c0_g1* encode a chlorophyll A/B binding protein, an autophagy protein (Cost1), and MYB-like DNA-binding domain, respectively. The up-regulated genes *TRINITY_DN20047_c0_g1*, *Zm00001d053091*, and *TRINITY_DN17398_c0_g1* encode an annexin, a flavin-binding kelch domain F box protein (FKF2) and a heat shock factor, respectively, except gene *TRINITY_DN73242_c0_g1* which encodes an unknown protein. The orthologs of the seven differentially expressed genes with function annotations were well documented for their response to extreme temperatures and other abiotic stresses [33,34,35,36,37,38,39,40,41,42,43,44,45,46,47,48].

Because drought and low-temperature stresses are major environmental factors that restrict maize growth and lead to yield decrease, the above results suggested that the transcriptional responses of many resistance-related genes were important mechanisms for 18-599 to adapt to the diverse ecological conditions in Southwest China [49,50].

## 4. Materials and Methods

### 4.1. Resequencing of Genomic DNA and Data Assembly

The genomic DNA of 18-599 was extracted with a QIAamp Tissue KitTM (Qiagen, Dusseldorf, Germany). After detection of concentration in Qubit 2.0 fluorometer (Invitrogen, Waltham, MA, USA), OD260/OD280 ratio in Nanodrop spectrophotometer (Thermo Fisher Scientific, Waltham, MA, USA), and integrity by 0.8% agarose gel electrophoresis, the qualified sample was sequenced on an Illumina Hiseq 2000 PE150 platform at Meiyin Gene (Beijing, China). Clean reads were filtered by removing adaptor contamination and low-quality reads (percentage of the low-quality bases of quality value ≤15 is more than 50% in a read, or percentage of unknown bases N > 1%) by FASTP [51] (https://www.biorxiv.org/content/, accessed on 10 March 2020), and mapped against the maize reference genome B73 (V4) (ftp://ftp.ensemblgenomes.org/pub/plants/release-38/fasta/zea_mays/dna/Zea_mays.AGPv4.dna.toplevel.fa.gz, accessed on 15 March 2020) by BWA software (http://bio-bwa.sourceforge.net/bwa.shtml, accessed on 15 March 2020) [52,53]. The results were indexed by SAMtools (http://samtools.sourceforge.net/, accessed on 24 March 2020) [54], and stored in BAM format. The duplicated reads were removed by MarkDuplicates (https://www.biostars.org/p/10019/, accessed on 26 March 2020). SNPs and InDels were called by Genome Analysis Toolkit (https://gatk.broadinstitute.org/hc/en-us, accessed on 10 April 2020) [55].

### 4.2. Identification of Specific Variation Loci and Phylogenetic Analysis

A Perl script was used to identify specific SNPs and InDels of 18-599 by alignment of the SNPs and InDels between 18-599 and the B73 genomic sequence, and those between the 1020 inbred lines in HapMap database v3.21 (https://www.sanger.ac.uk/resources/downloads/human/hapmap3.html, accessed on 20 April 2020) and the B73 genomic sequence [56]. The functions of the genes involving these SNPs and InDels were annotated by ANNOVAR (https://doc-openbio.readthedocs.io/projects/annovar/en/latest/, accessed on 10 May 2020). The genes with variation loci of significant impact on their function, such as frameshift, change of splicing site, stop gain, change of start site, and stop loss, were filtered. Inbred lines with an identity of SNPs and InDels more than 50% with 18-599 were filtered from the HapMap database v3.21. After removing deletion and heterozygous genotypes, they were used to construct a phylogenetic tree with 18-599 by PHYLIP (https://evolution.genetics.washington.edu/phylip.html, accessed on 16 June 2020).

### 4.3. RNA Preparation and Sequencing

In total, three independent biological replicates of 18-599 seedlings were stressed under simulative drought conditions of 16% (Polyethylene glycol, PEG), low temperatures of 8 °C 14 h/4 °C 10 h, and 28 °C and 300 mmol/m^2^·s (blank control), respectively. Three days later, each of the replicates was ground in liquid nitrogen under RNase-free conditions, and used for RNA extraction by using RNAiso Plus (TaKaRa, Dalian, China). Possible contamination of genomic DNA was removed by gDNA Eraser Kit (TaKaRa, Japan). After detection of concentration in Qubit 2.0 fluorometer (Invitrogen, USA), OD260/OD280 ratio in Nanodrop spectrophotometer (Thermo Fisher Scientific, USA), and integrity by 0.8% agarose gel electrophoresis, the qualified samples were sequenced on an Illumina Hiseq xten platform at Meiyin Gene (China). Evaluation of sequencing quality and filtration of clean reads were similar as described above. The clean reads were mapped against the maize reference genome sequence of B73 (V4), indexed by SAMtools (http://samtools.sourceforge.net/, accessed on 18 September 2020), and stored in BAM format. The unmapped reads were de novo assembled by TRINITY (https://www.plob.org/tag/trinity, accessed on 5 January 2021) [57]. The longest assembled result of the same TRINITY clustering was filtered by a Perl script (Supplement S1). The unigenes were indexed by HISAT2 v 2.1.0 (https://www.biostars.org/p/288726/, accessed on 12 March 2021), integrated with GTF annotation file of B73 genome (Zea_mays.AGPv4.38.gtf). The unmapped reads of each individual were mapped onto unigene sequences. The result of alignment was merged with the alignment of B73 for each sample.

### 4.4. Identification and Functional Annotation of Differentially Expressed Genes

The expression level of each individual was estimated by StringTie (http://ccb.jhu.edu/software/stringtie/, accessed on 20 April 2021) [58]. The results were converted by a Python script (http://ccb.jhu.edu/software/stringtie/dl/prepDE.py, accessed on 26 April 2021) written by the developer of StringTie, and identified for differentially expressed genes by DESeq 2 (https://www.bioconductor.org/packages/release/bioc/html/DESeq2.html, accessed on 16 May 2021) [59]. A Perl script (Supplement S2) was used to download coding sequences (CDS) from B73 genomic sequence B73_RefGen_V4 (ftp://ftp.ensemblgenomes.org/pub/plants/release-38/fasta/zea_mays/cds/Zea_mays.AGPv4.cds.all.fa.gz, accessed on 28 May 2021), Non-Redundant Protein Sequence (NR) Database (ftp://ftp.ncbi.nlm.nih.gov/blast/db/, accessed on 6 June 2021) and its annotation (ftp://ftp.ncbi.nlm.nih.gov/blast/db/FASTA/nr.gz, accessed on 13 June 2021), as well as Gene Ontology (GO) annotation (ftp://ftp.pir.georgetown.edu/databases/idmapping/idmapping.tb.gz, accessed on 22 June 2021). The longest CDS of each gene and the unigenes were used as query sequences to align against NR database by Diamond [60]. A Perl script (Supplement S3) was used to download GO annotation from idmapping.tb.gz—File Properties (ftp.pir.georgetown.edu, accessed on 6 July 2021). The functional enrichment of the differentially expressed genes under drought and low temperature was conducted using AgriGo database (http://systemsbiology.cau.edu.cn/agriGOv2/index.php, accessed on 25 July 2021) [61]. Graphical images of the significantly enriched GO terms were plotted using GGPLOT2 (http://ggplot.yhathq.com/, accessed on 10 August 2021).

### 4.5. RT-qPCR Verification

To verify the differential expression displayed by the above transcriptomics analysis, fifteen pairs of primers (Table S4) were designed by Primer-BLAST (https://www.ncbi.nlm.nih.gov/tools/primer-blast, accessed on 15 November 2021) and used for RT-qPCR amplification of fourteen random DEGs as well as internal control gene *ZmGAPDH*. The three replicates of the RNA samples prepared above were reversely transcribed into cDNA with TransStartR Tip Green qPCR SuperMix (Transgen Biotch, Beijing, China), and used as templates for two-step amplification of RT-qPCR by using SYBR^®^ Premix Ex Taq^TM^ II (Tli RNaseH Plus) (TaKaRa, Dalian, China) in CFX96^TM^ Real-Time System (Bio-Rad, Hercules, CA, USA). The temperature procedure was as follows: 95 °C for 30 s; 39 cycles of 95 °C for 5 s, and 50–57 °C for 30 s. At the end of the last cycle, the temperature was increased to 95 °C by 0.5 °C/s, so that a melting curve could be calculated and used to differentiate between specific and non-specific amplicons. The 2^−ΔΔCT^ method of the CFX Manger^TM^ software version 2.0 (Bio-Rad, Hercules, CA, USA) was used to normalize the expression differentiation between the sampled and the internal control genes [62]. The average relative expression levels of four technical and three biological replicates were calculated by their comparison to that of the internal control gene. The statistical significance was assessed via Student’s *t*-test with IBM-SPSS software (http://www-01.ibm.com/software/analytics/spss/, accessed on 26 February 2022).

## 5. Conclusions

Inbred line 18-599 not only pyramided the elite genes of P78599 but also acquired genetic divergence during the repetitive backcrosses of triple test cross, conferring its elite agronomic characteristics. The transcriptional responses of many resistance-related genes were important mechanisms for 18-599 to adapt to the diverse ecological conditions in Southwest China. However, a large number of the specific variations were found during the responsive DEGs and more in others. It was difficult to identify the direct connections between the specific variations and the responsive DEGs. The elite agronomic and adaptive traits remain to be elucidated, although they were thoroughly evaluated during the production practice. Ultimately, the results of this study still provide a reference for future research and breeding.

## Figures and Tables

**Table 1:** Specific variation loci with large impacts on gene functions in 18-599.

Variation Type	Number of Variation Loci	Number of Genes	Number of Variation Loci per Gene
Frameshift	1023	10,201	1.67
Change of splicing site	2293	1994	1.15
Stop gain	1879	1600	1.17
Change of start site	550	532	1.03
Stop loss	424	411	1.03
Total	21,961	12,297	1.79

**Table 2:** Inbred lines with identity of variation loci to 18-599 ≥50%.

Inbred Line	Identity	Inbred Line	Identity
ZEAxujRAUDIAAPE	73.65%	L005	54.40%
ZEAxppRDHDIAAPEI-12	60.11%	Shen 135	54.13%
ZEAxujRAVDIAAPE	59.99%	DH138	54.01%
CAU 178	59.82%	CT109	53.92%
dupl-178	59.82%	9058	53.83%
78599	59.78%	L-1	53.51%
D856	58.64%	Cheng 18	53.22%
2005-4	58.12%	P138	53.00%
Shen 137	58.01%	L069	52.53%
Shen 977	56.66%	Lo1125	52.12%
ZEAxujRAJDIBAPE	55.48%	Shan 89-1	52.08%
Dan 599	55.33%	D1051	51.80%
Zun 90110	55.28%	PN2	51.16%
68122	55.14%	R150	50.95%
ZEAxppRDQDIAAPEI-3	54.69%	SZ3	50.50%
Qi 319	54.41%		

## Data Availability

Not applicable.

## Supplementary Materials

The following supporting information can be downloaded at: https://www.mdpi.com/article/10.3390/plants11233242/s1, Table S1: Number of clean reads mapped to sequences of the reference genome; Table S2: Differentially expressed genes in response to drought stress; Table S3: Differentially expressed genes in response to low-temperature stress; Table S4: Primers for RT-qPCR.
