# Supplementary material for: Genomic Characteristics of Elite Maize Inbred Line 18-599 and Its Transcriptional Response to Drought and Low-Temperature Stresses
Source: Plants (Basel). 2022 Nov 25;11(23):3242. doi: 10.3390/plants11233242 (PMC9739999; doi:10.3390/plants11233242)
Supplement: Supplementary file 1 [file plants-11-03242-s001.zip › plants-2001102 supplementary/Table S1.pdf]

Table S1. Number of clean reads mapped to sequences of reference genome.

Treatment	Relicate	Clean read	Mapped one time （%）	Mapped more than one time （%）	Unmapped
Drought	I	67129352	52513630 (78.23%)	222512 (3.31%)	12393210 (18.46%)
	II	65382452	51180550 (78.28%)	2491578 (3.81%)	11710324 (17.91%)
	III	54511976	42593504 (78.14%)	1852082 (3.40%)	10066444 (18.47%)
Low temperature	I	56384548	43960098 (77.96%)	1950308 (3.46%)	10474142 (18.58%)
	II	61726740	48381582 (78.38%)	2131632 (3.45%)	11213526 (18.17%)
	III	65417140	51218480 (78.30%)	2364502 (3.61%)	11834158 (18.09%)
Control	I	48021670	32713152 (68.12%)	1349644 (2.81%)	13958874 (29.07%)
	II	61820870	42656922 (69.00%)	2195962 (3.55%)	16967986 (27.45%)
	III	56798602	39489760 (69.53%)	1876334 (3.30%)	15432508 (27.17%)
