# Supplementary material for: Genomic Characteristics of Elite Maize Inbred Line 18-599 and Its Transcriptional Response to Drought and Low-Temperature Stresses
Source: Plants (Basel). 2022 Nov 25;11(23):3242. doi: 10.3390/plants11233242 (PMC9739999; doi:10.3390/plants11233242)
Supplement: Supplementary file 1 [file plants-11-03242-s001.zip › plants-2001102 supplementary/Table S4.pdf]

**Table S4**. Primers for RT-qPCR.

| Primer | Sequence |
|---|---|
| ZmGAPDH-F | 5′-CCATCACTGCCACACAGAAAAC-3′ |
| ZmGAPDH-R | 5′-AGGAACACGGAAGGACATACCAG-3′ |
| TRINITY_DN20047_c0_g1-F | 5′-TGTGCACCAGTCTTCTGTGT-3′ |
| TRINITY_DN20047_c0_g1-R | 5′-GCGTGTGCTTGCTATTCCTG-3′ |
| TRINITY_DN43011_c0_g1-F | 5′-CATGCATGCTCCTGGCTAGT-3′ |
| TRINITY_DN43011_c0_g1-R | 5′-ATCCTCAACCTTCGGCTTGG-3′ |
| Zm00001d043044-F | 5′-TTTCGGCATTGGCAAAGTGG-3′ |
| Zm00001d043044-R | 5′-CCGAGCAAAGTAGGCCTCAA-3′ |
| TRINITY_DN13689_c0_g1-F | 5′-CAGCGGCTACCTACCTATGC-3′ |
| TRINITY_DN13689_c0_g1-R | 5′-CAAGAATTAGCCACGCACGG-3′ |
| TRINITY_DN16287_c0_g1-F | 5′-GAAATCCGGTCCCGATCGAC-3′ |
| TRINITY_DN16287_c0_g1-R | 5′-AAAGCCAAGGAGTCGGTCAC-3′ |
| TRINITY_DN17398_c0_g1-F | 5′-TTAATTGGCTCAGCGCCTCA-3′ |
| TRINITY_DN17398_c0_g1-R | 5′-GATAAGATGCCTCTGGCCCC-3′ |
| TRINITY_DN33619_c1_g1-F | 5′-ACTTTCCTCGCCGAAACCAA-3′ |
| TRINITY_DN33619_c1_g1-R | 5′-TTTGGTCGAAGGATGCGTGA-3′ |
| TRINITY_DN36046_c1_g3-F | 5′-GCACTTGTCTGAACTCCCGA-3′ |
| TRINITY_DN36046_c1_g3-R | 5′-ATACACAGGAAGCCGCACTC-3′ |
| TRINITY_DN63145_c0_g1-F | 5′-CCGCTGGGTCAAGTCTTACA-3′ |
| TRINITY_DN63145_c0_g1-R | 5′-AGGTAGCTGCATATGGCGTC-3′ |
| TRINITY_DN72435_c0_g1-F | 5′-CCTGGTCCATCTAGATAGGTGTT-3′ |
| TRINITY_DN72435_c0_g1-R | 5′-TCCTGACCAAGACGTTCGAC-3′ |
| TRINITY_DN73242_c0_g1-F | 5′-CAGTCAGCCCCTAAGACACG-3′ |
| TRINITY_DN73242_c0_g1-R | 5′-CAGGAGCGAGCCAAGAAAGA-3′ |
| Zm00001d053091-F | 5′-ATGGCTGGTGGTATTTGGGG-3′ |
| Zm00001d053091-R | 5′-GACCAGCTTCGAACCATCCA-3′ |
| TRINITY_DN39631_c1_g1-F | 5′-AGAACTGCAAGGGCAACAGA-3′ |
| TRINITY_DN39631_c1_g1-R | 5′-ACTTGGCAAAATGGGGGCTA-3′ |
